# Investigating Risk Factors for Racial Disparity in E-Cigarette Use with PATH Study

**DOI:** 10.3390/stats7030037

**Published:** 2024-06-21

**Authors:** Amy Liu, Kennedy Dorsey, Almetra Granger, Ty-Runet Bryant, Tung-Sung Tseng, Michael Celestin, Qingzhao Yu

**Affiliations:** 1 Statistics and Computer Science, Duke University, Trinity College of Arts and Sciences, Durham, NC 27708, USA; 2 Behavioral and Community Health Sciences, Louisiana State University Health Sciences Center School of Public Health, New Orleans, LA 70112, USA; 3 Biostatistics and Data Sciences, Louisiana State University Health Sciences Center School of Public Health, New Orleans, LA 70122, USA

**Keywords:** e-cigarettes, smoking cessation, disparities

## Abstract

**Background:**

Previous research has identified differences in e-cigarette use and socioeconomic factors between different racial groups However, there is little research examining specific risk factors contributing to the racial differences.

**Objective:**

This study sought to identify racial disparities in e-cigarette use and to determine risk factors that help explain these differences.

**Methods:**

We used Wave 5 (2018–2019) of the Adult Population Assessment of Tobacco and Health (PATH) Study. First, we conducted descriptive statistics of e-smoking across our risk factor variables. Next, we used multiple logistic regression to check the risk effects by adjusting all covariates. Finally, we conducted a mediation analysis to determine whether identified factors showed evidence of influencing the association between race and e-cigarette use. All analyses were performed in R or SAS. The R package mma was used for the mediation analysis.

**Results:**

Between Hispanic and non-Hispanic White populations, our potential risk factors collectively explain 17.5% of the racial difference, former cigarette smoking explains 7.6%, receiving e-cigarette advertising 2.6%, and perception of e-cigarette harm explains 27.8% of the racial difference. Between non-Hispanic Black and non-Hispanic White populations, former cigarette smoking, receiving e-cigarette advertising, and perception of e-cigarette harm explain 5.2%, 1.8%, and 6.8% of the racial difference, respectively. E-cigarette use is most prevalent in the non-Hispanic White population compared to non-Hispanic Black and Hispanic populations, which may be explained by former cigarette smoking, exposure to e-cigarette advertising, and e-cigarette harm perception.

**Conclusions:**

These findings suggest that racial differences in e-cigarette use may be reduced by increasing knowledge of the dangers associated with e-cigarette use and reducing exposure to e-cigarette advertisements. This comprehensive analysis of risk factors can be used to significantly guide smoking cessation efforts and address potential health burden disparities arising from differences in e-cigarette usage.

## 1. Introduction

The prevalence of e-cigarette use is growing in the United States. In a survey conducted in 2014–2015, 8.5% of the surveyees responded that they had tried e-cigarettes at least once [1]. E-cigarettes have long been advertised as smoking cessation tools [1]. Yet, there were studies that found that individuals who do use e-cigarettes have lower odds of quitting cigarette smoking compared to individuals who do not use them [2].

Additionally, significant health impacts were found to be associated with e-cigarette usage; for example, 2051 cases of lung injury were associated with e-cigarette usage in the United States as of 2019 [3]. However, studies have also found that e-cigarettes are lower in toxic contents compared to cigarettes (e.g., carcinogens, carbon monoxide, and oxidant gases), and that they have lower secondhand toxicity exposure as well, indicating different health burdens and effects of e-cigarettes and cigarettes [4,5].

Recently, many studies have analyzed the racial differences in e-cigarette usage. Non-Hispanic Whites were found to be more likely than non-Hispanic Blacks and Hispanics to become exclusive e-cigarette users [6-8]. Within the White population, a positive association between anxiety sensitivity and e-cigarette dependence was also found to be significant [8]. Additionally, White individuals were more likely to use e-cigarettes to save money compared to Hispanics [9].

In addition, Black e-cigarette users were found to be more likely to report plans to continue using e-cigarettes compared to their White and Hispanic counterparts, and Black participants were most likely to use e-cigarettes as a smoking cessation tool [9]. Other studies have found that Black and Hispanic smokers are more likely to have positive tobacco-related social norms [6]. Black and Asian populations are more likely to trust e-cigarette companies with information about the health effects of e-smoking compared to White communities [10]. Thus, the lower prevalence of e-cigarette usage in non-Hispanic Black and Hispanic communities is particularly interesting, and we sought to identify what factors we could learn from these communities in incorporating more robust and effective cessation practices.

Socioeconomic factors (income and education) were found to play a significant role in differences in e-cigarette use, such that adults with lower educational attainment were more likely to use more tobacco products, and low-income smokers were less likely to use e-cigarettes [6]. However, factors associated with lower tobacco use, such as higher socioeconomic (education and income) levels, are less protective for Blacks and Hispanics compared to their counterparts [11,12].

Although there has been a plethora of literature regarding the differences in e-cigarette and tobacco use, there is a severe lack of studies examining risk factors that may contribute to racial differences in e-cigarette usage. Therefore, our study sought to identify racial differences in e-cigarette usage and risk factors that help explain these differences.

## 2. Methods

### 2.1. Using PATH Data

The Population Assessment of Tobacco and Health (PATH) Study [13] is a national longitudinal study of tobacco use and its effect on people’s health (youth 12–17 years old and adults 18 years and older) in the United States. Wave 1 of the study was administered in 2013–2014, and respondents were followed in 2014–2015 (Wave 2), 2015–2016 (Wave 3), 2016–2018 (Wave 4), 2018–2019 (Wave 5), and 2021 (Wave 6). We used the public-use files of the PATHWave 5 (2018–2019) dataset.

### 2.2. Sample Selection Criteria

Our study sample included non-Hispanic White (n = 16,274), non-Hispanic Black (n = 4287), non-Hispanic Other (n = 2262), and Hispanic (n = 5250) individuals who were 18 years and older.

### 2.3. E-Cigarette Usage

The focal outcome was current, established e-cigarette usage, defined by the PATH Study as respondents “who have ever used any electronic nicotine products, have ever used them fairly regularly, and currently use every day or some days”.

### 2.4. Race-Ethnicity

The exposure variable of interest was race/ethnicity. Race-ethnicity is an accepted and established term, used by the Centers for Disease Control (CDC) and other agencies that release official statistics on population groups [14]. Race-ethnicity was categorized into non-Hispanic White, non-Hispanic Black, non-Hispanic Other, and Hispanic. Individuals who answered “Hispanic” to the survey for “Hispanic Origin” were categorized as Hispanic, with individuals answering “not Hispanic” categorized as non-Hispanic. Furthermore, we utilized the “recoded race” question in categorizing race-ethnicity, with individuals answering “White alone” being categorized into “non-Hispanic White” if they also answered “not Hispanic”; individuals answering “Black alone” being categorized into “non-Hispanic Black” if they also answered “not Hispanic”; and individuals answering “Other” being categorized into “non-Hispanic Other” if they answered “not Hispanic” to the Hispanic origin question.

### 2.5. E-Cigarette Smoking Risk Factor Variables

Risk factor variables that we were interested in exploring to determine whether they helped explain potential racial differences included total household income, education level, self-perception of mental health, perception of e-cigarette harm, social influences, former smoking, and reception of e-cigarette discounts or coupons.

Previous studies have found that attitudes towards e-cigarettes (e.g., believing that e-cigarettes are more harmful than cigarettes) and social influences (e.g., believing that most people disapprove of e-cigarette usage) contributed to decreased rates of e-cigarette uptake [6]. Additionally, Black e-cigarette users were found to be more likely to use e-cigarettes because of advertising [6].

Mental health has also been found to increase the hazard ratio of other tobacco initiation and progression amongst young adults, with those having poor and fair mental health being more likely to utilize water pipe smoking than those with good mental health [15].

Previous surveys have also found that the majority of e-cigarette users are current or former users who believe e-cigarettes can help them quit smoking or are less harmful than tobacco cigarettes [4].

Racial and ethnic minority adolescents have also been found to report higher engagement with tobacco marketing compared to their non-Hispanic white peers [16].

Table 1 shows the variables and how they are measured by the PATH survey questions.

PATH demographic survey data were analyzed using a 5-point Likert scale and categorized as follows: income less than USD 10,000, USD 10,000–24,999, USD 25,000–49,999, USD 50,000–99,999, and USD 100,000 or more; education level: “less than high school”, “GED”, “high school graduate”, “some college (no degree) or associate’s degree”, and “bachelor’s degree or more advanced degree”.

Self-perception of mental health was categorized into a 5-point Likert scale from poor to excellent.

The perception of e-cigarette harm was assessed by the survey prompt “General perception: Harmfulness of using e-cigarettes or other electronic nicotine products to health”, which was categorized into “not at all harmful”, “slightly harmful”, “somewhat harmful”, “very harmful”, and “extremely harmful”.

Social influences of e-cigarette smoking were assessed in the PATH survey by the question “People who are important to you use the following products: Cigarettes”, with answer choices of “yes” and “no”.

Former cigarette smoking status was divided into dichotomous categories of “Yes” (formerly smoked cigarettes) and “No” (did not formerly smoke cigarettes) in the survey. Former cigarette users were defined as “Wave 5 adult respondents who have smoked at least 100 cigarettes in their lifetime, and have not smoked them within the past 12 months or currently smoke them not at all”.

The PATH survey assessed the reception of e-cigarette advertising with the question “In the past 12 months, received discounts or coupons for any of the following products: E-cigarettes or other electronic nicotine products (including e-liquid)”, with answer choices of “yes” and “no”.

### 2.6. Statistical Analysis

All analyses were conducted via R in R Studio. All analyses were conducted with Wave 5 Cohort Single-Wave weights to adjust for the PATH survey design’s oversampling of tobacco users, young adults (aged 18–24), and Black adults [17].

We first conducted descriptive statistics of e-cigarette use across our risk factors (total household income, education level, self-perception of mental health, perception of e-cigarette harm, social influences, former smoking, and reception of e-cigarette discounts or coupons) and race variables separately. Next, we conducted chi-square tests to check the association between each risk factor variable and the e-cigarette usage outcome variable and between the race/ethnicity variable and the e-cigarette usage outcome variable to determine the significance of these factors in explaining e-cigarette use.

Next, we conducted multiple logistic regression to check the risk effects by adjusting all covariates. We utilized the “survey” [18] package in R to conduct descriptive statistics and multiple logistic regression tests.

**Figure 1.**
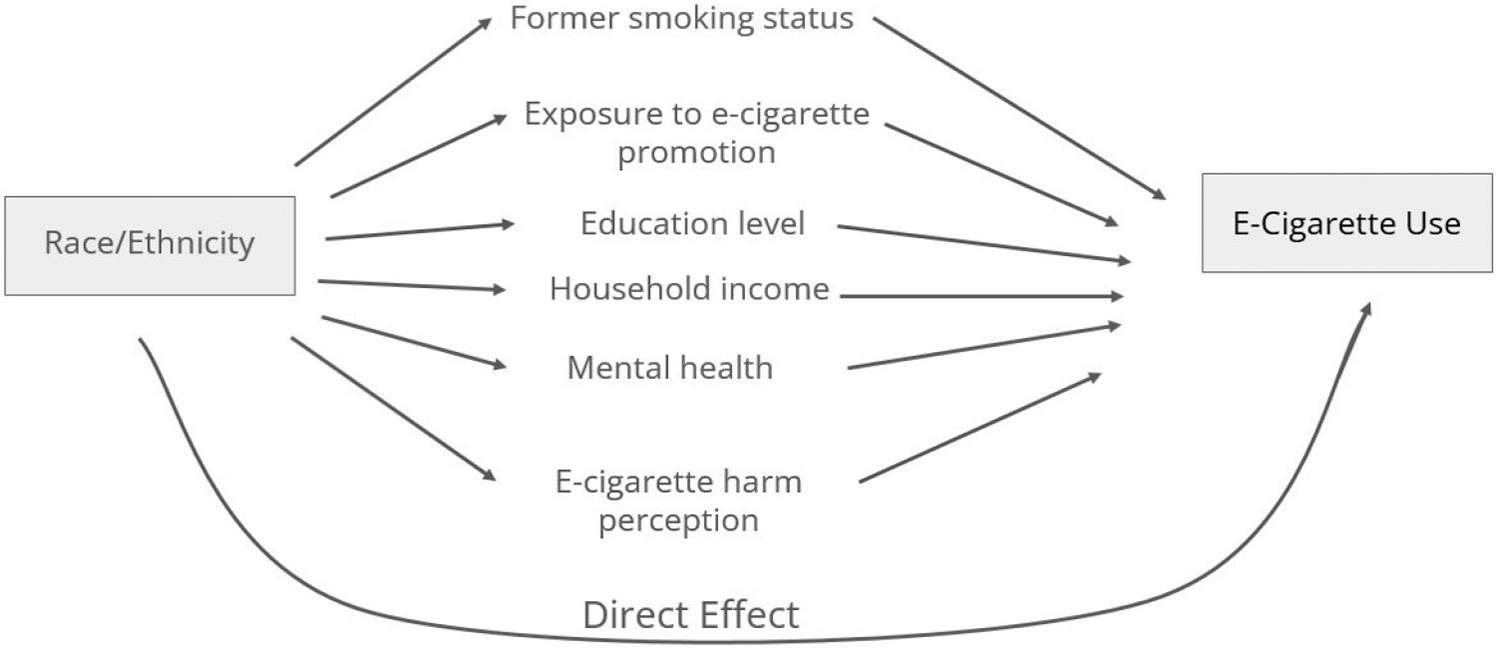
The conceptual model.

Finally, we utilized mediation analysis using the “mma” [19] package in R to determine whether the risk factors [20] showed evidence of influencing the association between race and e-cigarette use. In the R package, G-statistics were used to estimate the mediation effects, and the bootstrap method was used to find standard deviations and confidence intervals. The conceptual model is shown in Figure 1. We will make inferences on the effects from each path.

## 3. Results

### 3.1. Descriptive Analysis Results

Descriptive analysis using univariate analysis determined that in our dataset, non-Hispanic Whites had the greatest prevalence of e-cigarette usage (6.1%) compared with non-Hispanic Blacks (3.5%), Hispanics (3.6%), and non-Hispanic Others (5.1%) (Table 2).

We found that socioeconomic factors (total household income and education levels) were associated with different prevalence of e-cigarette usage (Table 3). Table 3 shows that the prevalence of e-cigarette usage decreased with the total household income level, and individuals with a bachelor’s or more advanced degree (2.4%) had the lowest prevalence of e-cigarette smoking compared to other education levels.

Individuals with more negative self-perception of mental health (e.g., categorizing their perception of their own mental health as “fair”, 8.7%, “poor”, 13.0%) were more likely to smoke e-cigarettes, especially compared to individuals with more positive self-perception of mental health (e.g., “excellent”, 4.1%, “very good”, 4.3%, “good”, 5.7%) (Table 3).

As expected, we found that individuals with lower levels of harm perception of e-cigarettes (e.g., perceiving cigarettes to be “not at all harmful”, 27.8%, or “slightly harmful”, 28.4%) were more likely to use e-cigarettes compared with those who answered with higher levels of harm perception (e.g., “somewhat harmful”, 9.5%, “very harmful”, 2.3%, “extremely harmful”, 1.5%) (Table 3).

Table 3 shows that individuals who had people important to them using e-cigarettes were more likely to smoke e-cigarettes (6.5%) compared to those who did not have people important to them smoking e-cigarettes (4.7%).

People who were former cigarette smokers (6.6%) were also found to have a higher prevalence of e-cigarette usage compared to people who did not formerly smoke cigarettes (5.0%) (Table 3).

Our descriptive analysis also found that individuals who received e-cigarette discounts or coupons were more likely to use e-cigarettes (20.1% vs. 4.9%) (Table 3).

Non-Hispanic Whites have the highest prevalence of being former cigarette smokers (14.2%), having people who are important to them use cigarettes (43.9%), and receiving e-cigarette discounts or coupons (4.9%) (Table 4).

Our descriptive analysis also determined that Non-Hispanic Blacks and Hispanics have the highest prevalence of making below USD 50,000 for total household income. Non-Hispanic Blacks and Hispanics also have a higher prevalence of perceiving e-cigarettes as “extremely harmful” (37.1%, 39.9%) (Table 4).

Non-Hispanic Blacks have the highest prevalence of obtaining education at the “high school” level (27%) (Table 4).

Table 4 also shows that non-Hispanic Whites and Hispanics are least likely to categorize their own mental health as “excellent” (23.6%, 23.8%).

### 3.2. Multiple Logistic Regression Results

In the multiple logistic regression model, the use of e-cigarettes was the binary outcome variable, and race/ethnicity, education, income, self-perception of mental health, e-cigarette harm perception, former cigarette usage, social influences, and reception of e-cigarette advertisements were the explanatory variables. We used SAS to fit the logistic regression model.

After adjusting for other risk factors, we found that non-Hispanic Whites are most likely to smoke e-cigarettes compared to non-Hispanic Blacks (Adjusted Odds Ratio (aOR): 0.47, *p* < 0.001) and Hispanics (*p* < 0.001) (aOR: 0.62). However, we did not find a significant difference in e-cigarette usage between non-Hispanic Whites and non-Hispanic Others (*p* = 0.095) (Table 5).

We also found that individuals with a bachelor’s or more advanced degree had the lowest odds of using e-cigarettes (aOR: 0.51, *p* < 0.001) compared to other education levels (Table 5). Table 5 shows that individuals with household incomes of USD 25,000–49,999 (aOR: 0.77, *p* = 0.021) and USD 50,000–99,999 (aOR: 0.79, *p* = 0.012) were less likely to smoke e-cigarettes than other household income levels.

Table 5 shows that significant differences in e-cigarette usage were found between individuals categorizing their own mental health as “excellent” or “good” and individuals categorizing their own mental health as “fair” (aOR: 1.62, *p* < 0.001). Significant e-cigarette usage differences were also found between individuals categorizing their own mental health as “excellent” or “good” and “poor” (aOR: 2.20, *p* < 0.001). For both, individuals with more negative perceptions of their mental health (“fair” or “poor”) were more likely to smoke e-cigarettes than individuals with positive perceptions of their mental health (“excellent”).

Compared to individuals who viewed e-cigarettes as “extremely harmful”, individuals with lower levels of harm perception of “slightly harmful” (aOR: 22.21, *p* < 0.001) and individuals who viewed e-cigarettes as “not at all harmful” (aOR: 22.49, *p* < 0.001) were significantly more likely to smoke e-cigarettes (Table 5).

Additionally, individuals who were former cigarette smokers had greater odds of e-cigarette usage (*p* < 0.001), as did individuals who received e-cigarette discounts and coupons (*p* < 0.001) (Table 5).

We found no significant difference in e-cigarette usage between individuals who answered “Yes” and individuals who answered “No” to the prompt “People who are important to you use cigarettes” (Table 5).

### 3.3. Mediation Analysis Results

The mediation analysis was performed using the “mma” package. The analysis was based on the “no-unmeasured confounding” assumptions [19,20].

The exposure variable in the mediation analysis was race-ethnicity, and the outcome variable was e-cigarette usage. Mediators are those variables that potentially explain the observed racial/ethnic difference in e-cigarette use. These variables included income, education, mental health, perception of e-cigarette harm, social influences, former cigarette smoking, and the reception of e-cigarette coupons or discounts. Note that the mediators may correlate with each other, but we assume there are no causal associations among the mediators.

We first performed a logistic regression to check whether there were racial/ethnic differences in the use of e-cigarettes without adjusting for any mediators. Table 6 shows that compared to non-Hispanic Whites, both the non-Hispanic Black (Odds Ratio (OR): 0.352, 95% Confidence Interval (C.I.): 0.295–0.414) and the Hispanic (OR: 0.553,95% C.I.: 0.483–0.63) populations were significantly (*p* < 0.05) less likely to use e-cigarettes. We did not find a significant difference in e-cigarette use between non-Hispanic White and non-Hispanic other populations.

Mediation analysis identified individual risk factors that significantly help explain differences in e-cigarette usage between both non-Hispanic Black and non-Hispanic White populations and between Hispanic and non-Hispanic White populations. We did not perform the mediation analysis between non-Hispanic whites and non-Hispanic others, since the two populations have no significant difference in using e-cigarettes (Table 6, *p*-value = 0.65). The risk factors included former cigarette smoking, receiving e-cigarette advertising, and perception of e-cigarette harm (Table 7).

**Figure 2.**
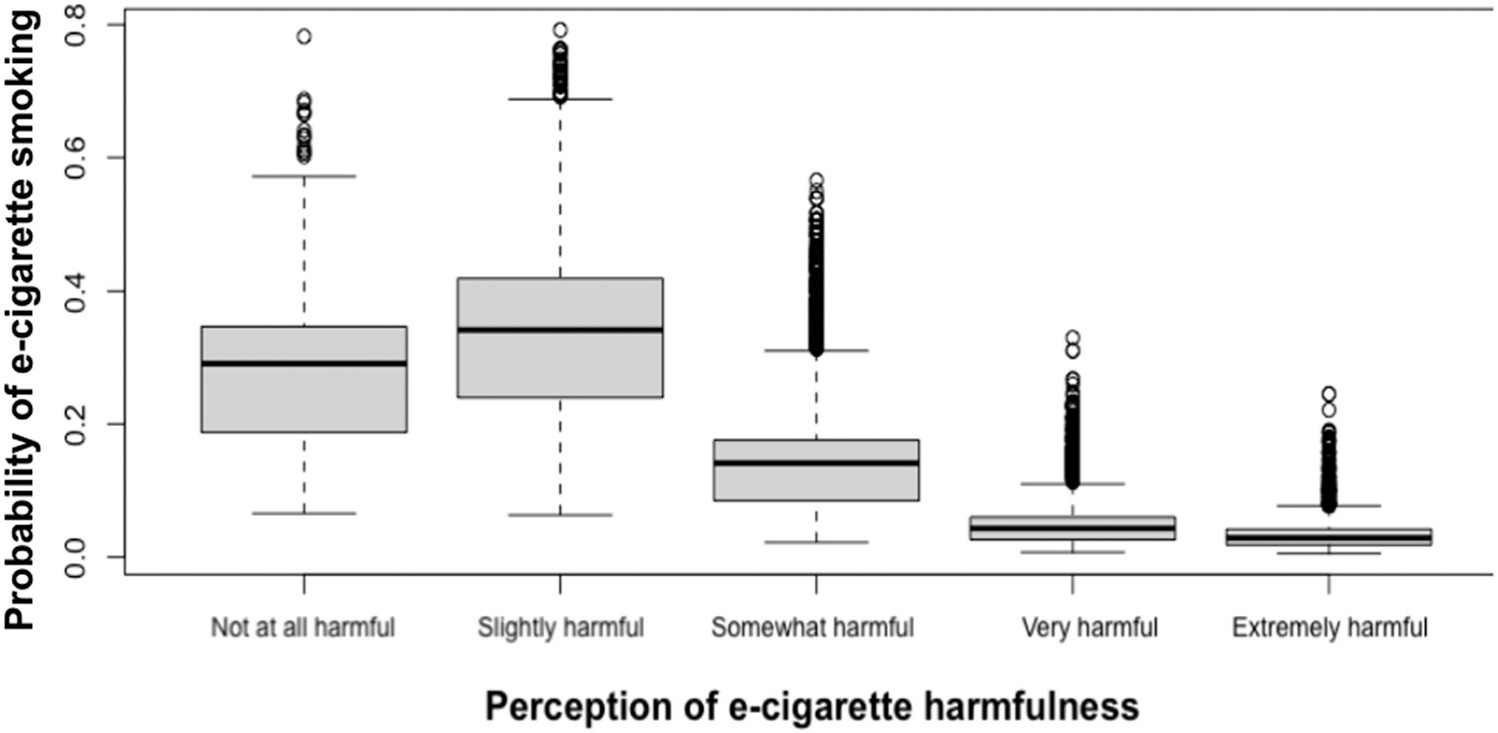
Probability of e-cigarette harmfulness perception categories to smoke e-cigarettes.

**Figure 3.**
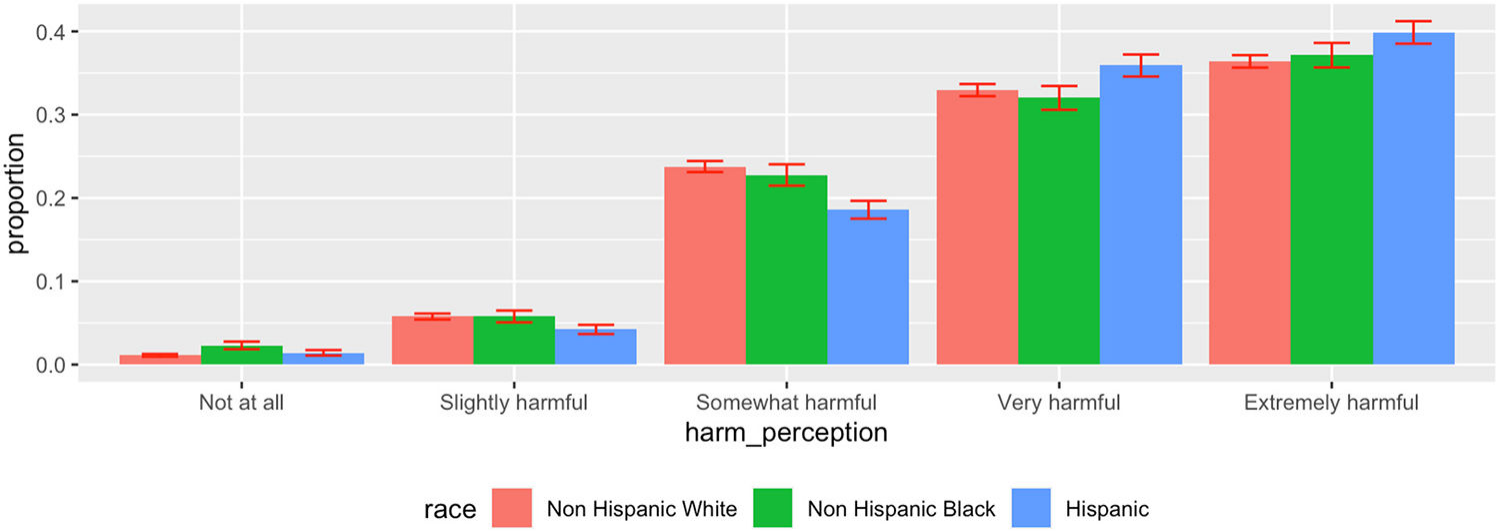
Comparison of e-cigarette harmfulness perception by race.

Table 7 shows the estimated relative effects through different risk factors. Relative effect is calculated as the (in)direct effect divided by the total effect. Based on the bootstrap samples, the confidence intervals were calculated using the 2.5% and 97.5% quantiles. Perception of e-cigarette harm helped explain 27.8% (95% C.I.: 21–36.4%) of the racial difference in e-cigarette smoking between Hispanics and non-Hispanic Whites and 6.8% (95% C.I.: 3.7–7.8%) of the racial difference in e-cigarette smoking between non-Hispanic Blacks and non-Hispanic Whites. To explain the mediation effect of the perception of e-cigarette harm, Figure 2 shows that the probability of e-cigarette smoking decreases with higher harm perception categories. Figure 3 shows that Hispanics, followed by non- Hispanic Blacks, have the highest proportion of perceiving the harm of e-cigarettes to be extremely harmful. Table 8 shows that on average, non-Hispanic Whites have a lower proportion of perceiving e-cigarettes to be “extremely harmful” compared to non-Hispanic Blacks and Hispanics.

**Figure 4.**
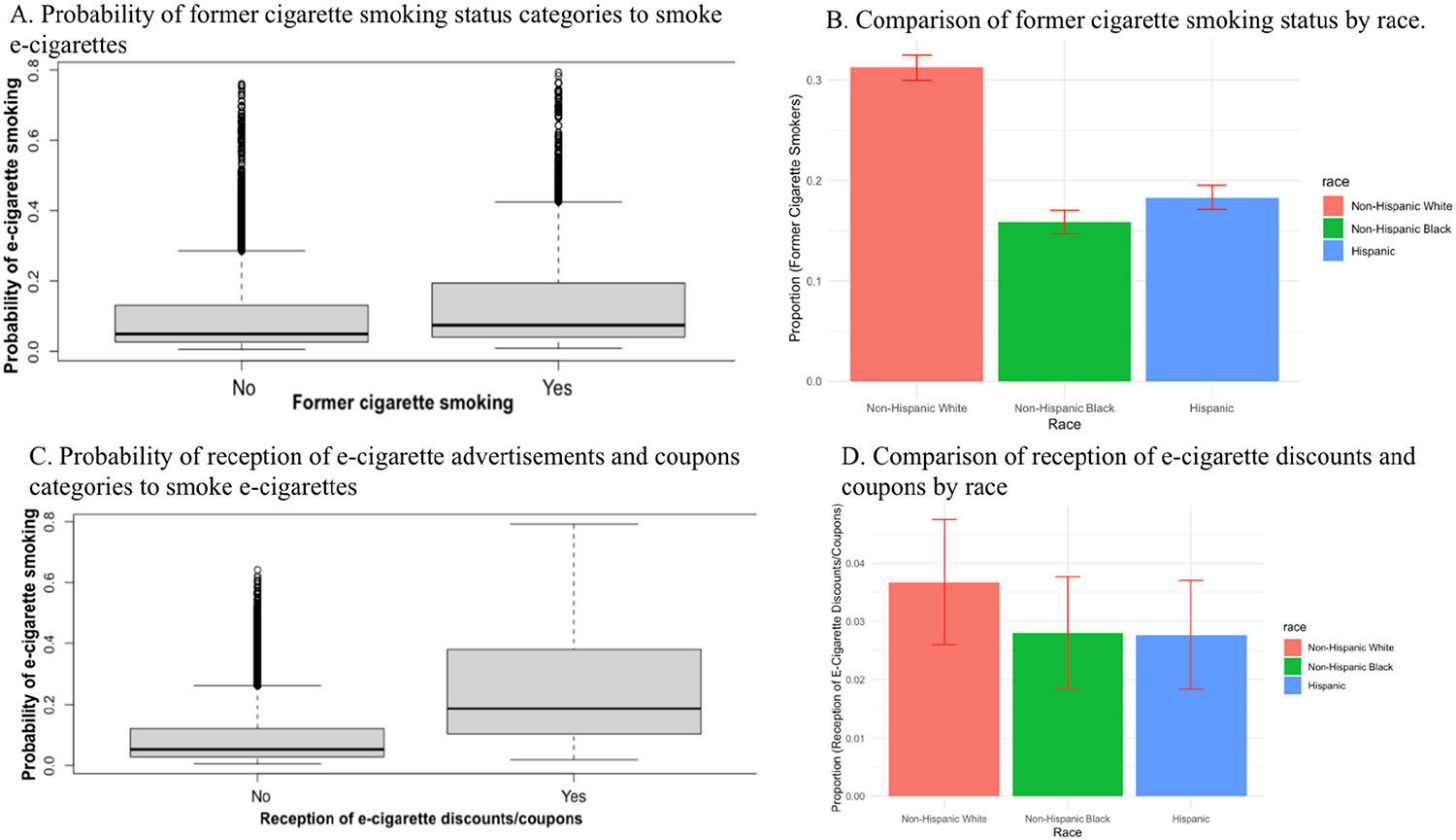
Former cigarette smoking and reception of e-cigarette discounts/coupons.

Former cigarette smoking status helped explain 7.6% (95% C.I.: 5.3–10.6%) of the racial difference in e-cigarette usage between Hispanics and non-Hispanic Whites and 5.2% (95% C.I.: 3.3–10.3%) of the racial difference in e-cigarette usage between non-Hispanic Blacks and non-Hispanic Whites. To explain this, the probability of e-cigarette usage increases for former cigarette smokers (Figure 4A), and non-Hispanic Whites had the highest prevalence of former cigarette smokers (Figure 4B). These differences in prevalence of former cigarette smokers were found to be significant (*p* < 0.05) between non-Hispanic White and non-Hispanic Black responses and non-Hispanic White and Hispanic responses (Figure 4B).

Receiving e-cigarette discounts and coupons helped explain 2.6% (95% C.I.: 1.2–4.3%) of the difference in e-cigarette use between Hispanics and non-Hispanic White populations and 1.8% (0.9–2.7%) of the difference in e-cigarette usage between non-Hispanic Blacks and non-Hispanic White populations. The probability of e-cigarette smoking increased for those who received these advertisements compared to those who did not (Figure 4C). Non-Hispanic Whites have the highest proportion of being exposed to these advertisements (Figure 4D). The differences in advertisement exposure were found to be significant (*p* < 0.05) between non-Hispanic White and non-Hispanic Black responses and between non-Hispanic White and Hispanic responses (Figure 4D).

The indirect effects of perception of e-cigarette harm, social influences, former smoking, and reception of e-cigarette discounts or coupons on the racial differences in e-cigarette smoking between Hispanic and non-Hispanic White populations were significant, with these factors collectively helping to explain 17.5% of the racial difference (95% C.I.: 8.8–26.9%).

## 4. Discussion

In this paper, we found that the odds of e-cigarette usage are highest in the non-Hispanic White population compared to non-Hispanic Black and Hispanic populations, and we did not find any significant difference in e-cigarette usage between non-Hispanic White and non-Hispanic Other populations. This is consistent with previous studies, which have found that the non-Hispanic White population is more likely to become exclusive e-cigarette users compared to the Hispanic and non-Hispanic Black populations [6-8].

Furthermore, we also observed that risk factors helping to explain these racial differences in e-cigarette usage were former cigarette smoking, receiving e-cigarette discounts and coupons, and perceived harm of e-cigarettes.

Additionally, we found that the risk factors of perception of e-cigarette harm, social influences, former smoking, and reception of e-cigarette discounts or coupons helped to explain roughly 17.5% of racial differences in e-cigarette usage between non-Hispanic White and Hispanic populations after adjusting for other variables. We also found that harm perception of e-cigarettes helped to explain 27.8% of racial differences in e-cigarette smoking between non-Hispanic White and Hispanic populations.

Through our multiple logistic regression, we found that harm perception plays a role in the e-cigarette smoking differences, with individuals who perceive e-cigarettes to be somewhat, very, or extremely harmful having lower odds of smoking e-cigarettes compared to those perceiving e-cigarettes to be not at all harmful. This supports previous studies that show that higher harm perceptions of e-cigarettes lead to lower usage rates [6]. We also found that former cigarette smokers are more likely to smoke e-cigarettes than individuals who did not previously smoke cigarettes (consistent with previous studies showing that the majority of e-cigarette users are former users believing that e-cigarettes can help with tobacco cessation, or that e-cigarettes are less harmful than cigarettes [4]), and that individuals who received coupons or discounts were more likely to use e-cigarettes than those who did not.

## 5. Conclusions

Consistent with previous studies [6], we found that socioeconomic differences contribute to differences in e-cigarette usage, where individuals with a bachelor’s or more advanced degree have an average lower odds of smoking e-cigarettes compared to individuals with lower education levels, and the odds of individuals making less than USD 10,000 in their total household income smoking e-cigarettes are lower than those of individuals making greater levels of income.

We found that individuals who perceived their own mental health to be in worse condition (e.g., “poor”, “fair”) were more likely to use e-cigarettes compared to those who rated their own mental health as “excellent”. This supports previous studies’ findings that tobacco usage is correlated with lower mental health perception, as found in an increased usage of waterpipe smoking among young adults with poorer mental health perception [15].

Potential limitations of this study include the PATH Study’s design, in which the data may be affected by subjects’ response bias. Additionally, it may be helpful to analyze Wave 6 PATH data in future studies to determine whether COVID-19 has caused any shifts in smoking patterns, particularly with e-cigarette usage [21].

Another limitation is our sole focus on the PATH Study’s data, which allows us to analyze only variables collected by the survey. Other risk factors not included in the PATH Study may contribute to racial/ethnic disparities.

Despite these limitations, this study has important implications. Although previous studies have examined the demographic characteristics of e-cigarette users and the prevalence of e-cigarette smoking across different races, few studies have analyzed the causal pathway between risk factors and e-cigarette smoking differences between populations. This study allows us to glean valuable insights regarding what we can learn from these communities to decrease the prevalence of e-cigarette usage overall. Our findings suggest that e-cigarette usage may be mitigated by increasing knowledge around the risks of e-cigarette usage, reducing e-cigarette advertisement exposure, and deconstructing the myth of e-cigarettes being useful cigarette cessation tools [2].

Additionally, this study’s findings will allow policymakers to determine how to effectively enable tobacco cessation efforts for each population and allow them to better assess the health impacts of tobacco use in their communities, as previous studies have found that smoking e-cigarettes and smoking cigarettes lead to different health impacts and burdens, sparking concern for increasingly widening health disparities in minority communities that come with these racial differences in tobacco usage.

## Figures and Tables

**Table 1.** Risk factor variables.

Variable Name	PATH Question	Categories
Total household income	Recoded total household income in the past 12 months	Less than USD 10,000 USD 10,000–24,999 USD 25,000–49,999 USD 50,000–99,999 USD 100,000 or more
Education level	Recoded education level	Less than high school GED High school graduate Some college (no degree) or associates degree Bachelor’s or more advanced degree
Self-perception of mental health	Self-perception of mental health	Excellent Very Good Good Fair Poor
Perception of e-cigarette harm	General perception: harmfulness of using e-cigarettes or other electronic nicotine products to health	Not at all harmful Slightly harmful Somewhat harmful Very harmful Extremely harmful
Social influences of e-cigarette smoking	People who are important to you use the following products: cigarettes	Yes No
Former cigarette smoking status	Wave 5 adult respondents who have smoked at least 100 cigarettes in their lifetime and who have not smoked them within the past 12 months or currently do not smoke them at all	Yes No
E-cigarette advertising	In the past 12 months, received discounts or coupons for any of the following products: e-cigarettes or other electronic nicotine products (including e-liquid)	Yes No

**Table 2.** E-Cigarette usage prevalence across race.

		Uses E-Cigarettes				
		Yes (freq)	Yes (%)	No (freq)	No (%)	*p*-Value
Race	Non-Hispanic White	8,028,170	6.1%	122,187,872	93.8%	*p* < 0.001
	Non-Hispanic Black	802,207	3.5%	21,765,158	96.4%	
	Non-Hispanic Other	829,856	5.1%	15,513,920	94.9%	
	Hispanic	1,029,934	3.6%	27,374,704	96.4%	

**Table 3.** E-cigarette usage prevalence across risk factor variables.

				Uses E-Cigarettes		
		Yes (freq)	Yes (%)	No (freq)	No	*p*-Value
**Income**	USD 10,000 and under	1,515,935	7.4%	18,991,401	92.6%	*p* < 0.001
	USD 10,000 to 24,999	2,308,847	6.8%	31,819,426	93.2%	
	USD 25,000 to 49,999	2,387,311	5.4%	41,721,298	94.6%	
	USD 50,000 to 99,999	2,648,910	5.0%	50,425,112	95.0%	
	USD 100,000 or more	1,829,165	4.0%	43,884,412	96.0%	
**Education level**	Less than high school	995,916	5.2%	18,008,815	94.8%	*p* < 0.001
	GED	812,566	8.0%	9,350,832	92.0%	
	High school graduate	2,951,174	6.7%	41,101,620	93.3%	
	Some college (no degree) or associates degree	4,473,799	7.1%	58,237,944	92.9%	
	Bachelor’s degree or advanced degree	1,456,712	2.4%	60,142,439	97.6%	
**Mental health**	Excellent	2,029,028	4.1%	47,502,744	95.9%	*p* < 0.001
	Very good	2,803,241	4.3%	63,027,358	95.7%	
	Good	2,956,205	5.7%	49,098,726	94.3%	
	Fair	2,062,095	8.7%	21,604,072	91.3%	
	Poor	839,599	13.0%	5,608,749	87.0%	
**Perception of e-cigarette harmfulness**	Not at all harmful	710,298	27.8%	1,846,255	72.2%	*p* < 0.001
	Slightly harmful	3,119,688	28.4%	7,879,438	71.6%	
	Somewhat harmful	4,262,247	9.5%	40,813,707	90.5%	
	Very harmful	1,514,805	2.3%	64,551,951	97.7%	
	Extremely harmful	1,083,130	1.5%	71,750,298	98.5%	
**People who are important to you use cigarettes**	Yes	5,189,892	6.5%	5,500,276	93.5%	*p* < 0.001
	No	74,390,073	4.7%	112,451,576	95.3%	
**Former smoker**	Yes	3,517,413	6.6%	49,194,303	93.3%	*p* < 0.001
	No	7,172,755	5.0%	137,647,346	95.0%	
**Received e-cigarette coupons or discounts**	Yes	1,333,243	20.1%	5,285,811	79.9%	*p* < 0.001
	No	9,356,925	4.9%	181,555,839	95.1%	

**Table 4.** Distribution of risk factors across races.

		Race				
		Non-Hispanic White (n = 130,216,042)	Non-Hispanic Black (n = 22,567,361)	Non-Hispanic Other (n = 16,343,776)	Hispanic (n = 28,404,638)	*p*-Value
**Income**	USD 10,000 and under	6.8	22.4	7.2	18.9	*p* < 0.001
	USD 10,000 to 24,999	14.9	23.3	13.5	25.5	
	USD 25,000 to 49,999	21.1	24.2	21.7	26.9	
	USD 50,000 to 99,999	30.0	19.9	23.8	20.0	
	USD 100,000 or more	27.2	10.2	33.8	8.7	
**Education level**	Less than high school	6.5	11.6	5.1	25.0	*p* < 0.001
	GED	4.8	5.7	1.9	8.3	
	High school graduate	21.7	27.0	14.9	25.8	
	Some college (no degree)or associate’s degree	32.4	36.6	27.6	27.2	
	Bachelor’s degree or advanced degree	34.7	19.1	50.4	13.8	
**Mental health**	Excellent	23.6	31.1	30.9	2.38	*p* < 0.001
	Very good	35.3	28.3	32.8	28.4	
	Good	26.0	25.4	24.1	30.2	
	Fair	11.9	12.0	8.8	14.3	
	Poor	3.3	3.1	3.4	3.3	
**Perception of e-cigarette harmfulness**	Not at all harmful	1.1	2.3	1.1	1.4	*p* < 0.001
	Slightly harmful	5.8	5.8	6.0	4.2	
	Somewhat harmful	23.8	22.8	22.7	18.6	
	Very harmful	33.0	32.0	35.1	35.9	
	Extremely harmful	36.4	37.1	35.1	39.9	
**People who are important to you use cigarettes**	Yes	43.9	40.6	30.7	29.0	*p* < 0.001
	No	56.1	59.4	69.3	71.0	
**Former cigarette smoker**	Yes	31.2	15.6	18.3	19.3	*p* < 0.001
	No	68.8	84.4	81.7	80.7	
**Received e-cigarette coupons or discounts**	Yes	4.9	3.3	4.6	3.5	*p* = 0.006
	No	95.1	96.7	95.4	96.5	

Note: Each cell reflects percentages.

**Table 5.** Multiple logistic regression Adjusted Odds Ratios (aOR) of e-cigarette usage.

		aOR	*p*-Value
**Race**	Non-Hispanic White	(ref)	(ref)
	Non-Hispanic Black	**0.47** (0.37, 0.61)	<0.001
	Non-Hispanic Other	0.99 (0.81, 1.22)	0.095
	Hispanic	**0.61** (0.49, 0.78)	<0.001
**Income**	USD 10,000 and under	(ref)	(ref)
	USD 10,000 to 24,999	0.90 (0.72, 1.12)	0.353
	USD 25,000 to 49,999	**0.77** (0.61, 0.96)	0.021
	USD 50,000 to 99,999	**0.79** (0.65, 0.95)	0.012
	USD 100,000 or more	0.86 (0.67, 1.10)	0.220
**Education**	Less than HS	(ref)	(ref)
	GED	**1.36** (1.02, 1.81)	0.032
	HS graduate	**1.28** (1.08, 1.53)	0.006
	Some college/associates	**1.36** (1.11, 1.66)	0.003
	Bachelors/above	**0.51** (0.40, 0.67)	<0.001
**Mental health**	Excellent, very good	(ref)	(ref)
	Good	1.14(0.98, 1.32)	0.098
	Fair	**1.62 (1.38, 1.92)**	<0.001
	Poor	**2.20 (1.80, 2.70)**	<0.001
**Perception of e-cigarette harmfulness**	Extremely harmful	(ref)	
	Very harmful	1.48 (1.24, 1.77)	<0.001
	Somewhat harmful	6.15 (5.21, 7.27)	<0.001
	Slightly harmful	22.21 (18.22, 27.08)	<0.001
	Not at all harmful	22.49 (16.59, 30.47)	<0.001
**People who are important to you use cigarettes**	No	(ref)	(ref)
	Yes	1.05 (0.94, 1.17)	0.422
**Former cigarette smoker**	No	(ref)	(ref)
	Yes	**1.45** (1.28, 1.63)	<0.001
**Received e-cigarette coupons or discounts**	No	(ref)	(ref)
	Yes	**3.73** (3.09, 4.50)	<0.001

**Table 6.** Racial/ethnic differences in e-cigarette usage.

		Adjusted Odds Ratio (Confidence Interval) aOR (95% C.I.)	*p*-Value
Race	Non-Hispanic White	(ref)	(ref)
	Non-Hispanic Black	**0.35 (0.30–0.41)**	<0.001
	Non-Hispanic Other	0.96 (0.81–1.13)	0.650
	Hispanic	**0.55 (0.48–0.63)**	<0.001

**Table 7.** Relative effects of risk factors upon e-cigarette usage.

	Risk Factor	Relative Effect (95% C.I.)	Standard Error
Non-Hispanic White vs. Non-Hispanic Black	Direct effect:	**0.961 (0.929, 1.015)**	0.030
	Former cigarette smoking	**0.052 (0.037, 0.068)**	0.008
	Receiving e-cigarette coupons or discounts	**0.018 (0.009, 0.027)**	0.004
	Education level	−0.129 (−0.166, 0.098)	0.017
	Total household income	0.011 (−0.022, 0.045)	0.017
	Mental health	0.002 (−0.01, 0.014)	0.006
	E-cigarette harm perception	**0.068 (0.033, 0.103)**	0.018
Non-Hispanic White vs. Hispanic	Direct effect:	**0.807 (0.749, 0.882)**	0.047
	Former cigarette smoking	**0.076 (0.053, 0.106)**	0.014
	Receiving e-cigarette coupons or discounts	**0.026 (0.012, 0.043)**	0.008
	Education level	−0.226 (−0.31, −0.16)	0.039
	Total household income	0.026 (−0.017, 0.07)	0.022
	Mental health	−0.004 (−0.019, 0.01)	0.007
	E-cigarette harm perception	**0.278 (0.21, 0.364)**	0.039

**Table 8.** E-cigarette harmfulness perception category differences by race.

Non-Hispanic White vs. Non-Hispanic Black (“not at all”):	Non-Hispanic White vs. Non-Hispanic Black (“slightly”):	Non-Hispanic White vs. Non-Hispanic Black (“somewhat harmful”):	Non-Hispanic White vs. Non-Hispanic Black (“very harmful”):	Non-Hispanic White vs. Non-Hispanic Black (“extremely harmful”):
C.I.: (−0.0119, −0.0118), *p* < 0.001	C.I.: (−2.11 × 10^−4^, −3.36 × 10^−6^), *p* < 0.05	C.I.: (0.009, 0.01), *p* < 0.001	C.I.: (0.0091, 0.0096), *p* < 0.001	C.I.: (−0.0077, −0.0072), *p* < 0.001
Non-Hispanic White vs. Hispanic (“not at all”):	Non-Hispanic White vs. Hispanic (“slightly”):	Non-Hispanic White vs. Hispanic (“somewhat harmful”):	Non-Hispanic White vs. Hispanic (“very harmful”):	Non-Hispanic White vs. Hispanic (“extremely harmful”):
C.I.: (−0.0119, −0.0118), *p* < 0.001	C.I.: (0.0154, 0.0155), *p* < 0.001	C.I.: (0.0517, 0.052), *p* < 0.001	C.I.: (−0.0297, −0.0293), *p* < 0.001	C.I.: (−0.0349, −0.0346), *p* < 0.001

## Data Availability

We used restricted access to the PATH dataset. Researchers can request access to the dataset through the website. https://pathstudyinfo.nih.gov/.
